# The neutralizing role of IgM during early Chikungunya virus infection

**DOI:** 10.1371/journal.pone.0171989

**Published:** 2017-02-09

**Authors:** Chong-Long Chua, I-Ching Sam, Chun-Wei Chiam, Yoke-Fun Chan

**Affiliations:** Department of Medical Microbiology, Faculty of Medicine, University of Malaya, Kuala Lumpur, Malaysia; CEA, FRANCE

## Abstract

The antibody isotype IgM appears earlier than IgG, within days of onset of symptoms, and is important during the early stages of the adaptive immune response. Little is known about the functional role of IgM during infection with chikungunya virus (CHIKV), a recently reemerging arbovirus that has caused large global outbreaks. In this study, we studied antibody responses in 102 serum samples collected during CHIKV outbreaks in Malaysia. We described the neutralizing role of IgM at different times post-infection and examined the independent contributions of IgM and IgG towards the neutralizing capacity of human immune sera during the early phase of infection, including the differences in targets of neutralizing epitopes. Neutralizing IgM starts to appear as early as day 4 of symptoms, and their appearance from day 6 is associated with a reduction in viremia. IgM acts in a complementary manner with the early IgG, but plays the main neutralizing role up to a point between days 4 and 10 which varies between individuals. After this point, total neutralizing capacity is attributable almost entirely to the robust neutralizing IgG response. IgM preferentially binds and targets epitopes on the CHIKV surface E1-E2 glycoproteins, rather than individual E1 or E2. These findings provide insight into the early antibody responses to CHIKV, and have implications for design of diagnostic serological assays.

## Introduction

Chikungunya virus (CHIKV) is an alphavirus from the family *Togaviridae*. Its emergence has led to unprecedented global epidemics among immunological naïve populations across different countries in Asia, the Americas, Africa and Europe [1]. *Aedes* mosquitoes serve as the main vectors in disease transmission. Phylogenetic analysis reveals the existence of three distinct CHIKV genotypes: West African, East/ Central/ South African (ECSA) and Asian, with the ECSA genotype causing the recent epidemics in India, the Indian Ocean and Southeast Asia, while the Asian genotype is responsible for the recent extensive outbreaks in the Americas and the Caribbean.

Infection of CHIKV is characterized by abrupt fever, profound acute joint pain, myalgia and erythematous maculopapular rashes [2, 3]. Other less specific symptoms include nausea and abdominal pain [4]. Viral loads of up to 10^9^ viral RNA copies per ml occur during early infection, and viremia may last for 5–7 days [5, 6]. Interferon type I, particularly interferon-alpha (IFN-α) is induced during the viremic period, and concentrations correlate with viral loads [7, 8]. Generally, IgM is detectable from day 3 to day 8 onwards after the onset of clinical symptoms, while convalescent IgG with neutralizing activity is produced from day 4 [9]. CHIKV is usually a self-limiting disease, with humoral immunity playing the pivotal role in control of infection and rapid virus clearance within days; nevertheless, debilitating arthralgia that mainly affects the small joints may persist for longer periods [10].

Pre-clinical studies on mouse models have shown the importance of antibody-mediated immunity in controlling infection [11, 12]. CHIKV infection of Rag1^-/-^ or Rag2^-/-^ (lacking mature lymphocytes) and μMT (B-cell deficient) mice resulted in persistent viremia accompanied by joint inflammation [11, 13, 14]. Passive transfer of CHIKV-specific antibodies into infected mice had both prophylactic and therapeutic effects [15]. Immune IgG from convalescent patients directly neutralizes CHIKV, and may persist in immune individuals for life [16, 17].

The functional role of infection-induced specific IgM against CHIKV is less well-characterized compared to immune IgG during acute and early convalescent phases of infection in mice and humans. Infection of athymic mice with the closely related alphavirus Semliki Forest virus revealed the role of IgM in clearing viremia, but not virus localized in the brain [18]. Induction of a specific, neutralizing IgM response by the flavivirus West Nile virus in mice reduces viremia and dissemination into the brain and spinal cord [19]. Similar observations were reported for rabies virus, influenza virus, vesicular stomatitis virus and smallpox vaccine, which demonstrated that induced IgM is important to confer protection, particularly in early stages before the IgG response [20–23]. A recent study in uninfected mice also demonstrated an unexpected role for natural antibodies, which are constantly secreted without specific stimulation as part of primary defence, in partially neutralizing CHIKV [11]. Natural antibodies limit early viral and bacterial dissemination, enhance antigen trapping in secondary lymphoid organs, and bridge innate and adaptive immunity [24, 25].

We hypothesized that IgM is important to provide early immunoprotection (particularly neutralizing capacity) prior to appearance of the full IgG response. The objectives of this study were to assess the function of immune (infection-induced) IgM in CHIKV neutralization, and to compare the contribution of IgM and IgG towards neutralizing capacity of human immune sera. We found that neutralizing IgM starts to appear as early as day 4 after disease onset and its appearance is associated with a reduction of viremia starting from day 6. IgM has the dominant neutralizing role up to day 10, with variable but strong contributions by neutralizing IgG. The neutralizing IgM preferably targets epitopes on the CHIKV surface E1-E2 glycoproteins.

## Materials and methods

### Ethical approval

This study was approved by the Medical Ethics Committee of the University Malaya Medical Centre (reference no. 20157–1467). Our institution does not require informed consent for retrospective studies of archived and anonymized samples.

### CHIKV immune serum panels

This study used two panels of serum samples. Panel A comprised 27 samples collected from patients attending University Malaya Medical Centre, Kuala Lumpur, during the 2008–2010 outbreak of CHIKV of East Central/ South African (ECSA) genotype. These were acute samples collected from viremic patients between day 1 and day 9 after disease onset. Viral loads had been quantified by real-time PCR targeting the E1 region in a previous study [26]. In this study, for all but 4 of the samples in this panel, only neutralizing titers of total antibodies (and not IgM and IgG separately) were determined due to limited sample volumes. This panel provided information regarding the relationship between viremia and the appearance of neutralizing antibodies.

For panel B, the neutralizing titers of IgM and IgG were determined separately in 79 samples to study the relative contributions to total neutralizing activity. Of these 79 samples, 39 serum samples were from the same 2008–2010 outbreak and known to contain neutralizing anti-CHIKV IgM and/or IgG [27]. These samples were collected from patients attending University Malaya Medical Centre 4 days to 6 months after symptoms, and included 4 samples from panel A for which sufficient serum volumes were available for additional analysis. A further 40 samples were collected from patients 11–14 months after an Asian CHIKV outbreak in Bagan Panchor, Perak state, in 2006 [28, 29]. The samples were categorized into 3 groups by the duration between sample collection and time of acute disease onset: 4–20 days (panel B1, n = 16), 1–6 months (panel B2, n = 23) and 11–14 months (panel B3, n = 40).

Serum samples from 15 healthy controls with no past infection of CHIKV were included as negative controls, confirmed by the absence of antibodies by serum neutralization assay. This made a total of 117 sera used in this study.

### Cells and viruses

Baby hamster kidney (BHK-21) cells (ATCC no. CCL-10) were maintained in Glasgow minimum essential medium (GMEM) (Life Technologies, USA) supplemented with 5% heat-inactivated fetal bovine serum (Bovogen Biologicals, Australia), 10% Tryptose phosphate broth (Sigma-Aldrich, USA), 20 mM HEPES, 5mM L-glutamine, 100 U/ml penicillin and 100 μg/ml streptomycin (Life Technologies). Infected cells were maintained in GMEM containing 2% FBS.

The virus strain used was MY/08/065 (GenBank accession number FN295485) at passage 3, a previously characterized ECSA virus isolated from a patient in Malaysia in 2008 [30]. The virus was propagated in BHK-21 cells and titrated by standard plaque assay. To study the neutralizing epitopes of anti-CHIKV IgM and IgG, CHIKV and previously constructed chimeric viruses carrying zsGreen reporter were rescued from infectious clones, as previously described [27]. The CHIKV infectious clone was derived from the ECSA genotype, based on LR2006-OPY1 and termed ICRES1 [31], and the chimeric viruses had the ecto-domain regions of envelope glycoproteins in the ICRES1 backbone replaced with those of Semliki Forest virus (SFV), a related alphavirus [27]. The chimera with E1 swapped from SFV was non-viable.

### Whole virus antigens and recombinant proteins

Two types of virus antigens were used for this study. For Western blot and indirect IgG ELISA, the antigen was partially purified virus prepared by sucrose-cushion ultra-centrifugation. Virus pellet was treated with 1% Triton X-100 in TE buffer, clarified by centrifugation and stored in the presence of 50% glycerol at -20°C. For capture IgM ELISA, formalin-treated virus supernatant was utilized as antigen. Formalin (37%) (Merck, Germany, cat. no. 1040032500) was added to a final concentration of 0.75%, with constant rotation at 4°C for 24 hours. This source of antigen was used within 3 days when kept at 4°C.

Standard molecular cloning was performed to clone E1 glycoprotein (rE1, from amino acids 1–412) and E2 glycoprotein (rE2, from amino acids 1–362), without the transmembrane and cytoplasmic tail regions, into a pIEX-5 vector (Novagen, USA). Fusion rE1 and rE2 was generated by overlapping PCR to link both fragments with a short linker GGGS-His (8X)-GGGG. All the constructs were transfected into TriExSf9 cells (Novagen) by TransIT-Insect Transfection Reagent (Mirus Bio, USA) [32]. The recombinant proteins were derived from MY/08/065 amino acid sequences.

### Enzyme-Linked Immunosorbent Assay (ELISA)

A capture IgM ELISA format was developed to determine the presence of anti-CHIKV IgM. All the incubation steps were performed at 37°C for 1 hour, using 1% BSA-0.05% PBST as diluents for serum and antibodies. The plates were washed 4 times with 0.05% PBST after each incubation step, and 6 times after antigen (formalin-treated virus supernatant), and monoclonal and secondary antibody incubation steps. The plate was coated with rabbit anti-human IgM (Dako Cytomation, Denmark, cat. no. A0425) to a final concentration of 2.8 μg/ml and blocked with 3% BSA-0.05% PBST. Sera were diluted at 1:200 and added. Antigen was then added, and this was either 10^6^ pfu per well of formalin-treated virus supernatant diluted in 1% BSA-PBS, or purified recombinant rE1 or rE2 glycoprotein diluted in 1% BSA-PBST to a final concentration of 20 μg/ml. Anti-CHIKV antibody was diluted in 1% BSA-PBST to a final concentration of 1 μg/ml and added to the plate, which was then incubated for 30 min. For the ELISA using rE2 and formalin-treated virus as antigens, anti-E2 monoclonal antibody B-D2(C4) [33] was the antibody used; for the ELISA using rE1 as antigen, anti-alphavirus antibody (Santa Cruz Biotechnology, USA, cat. no. sc-58088) detecting E1 [34] was used. Goat anti-mouse IgG-HRP (Bio-Rad, USA, cat. no. 170–6516) was added at 1:20,000 dilution, followed by 30 min incubation. TMB substrate (KPL) was added to each well and the plate was incubated at room temperature for 5 min. The reaction was terminated by adding 1M phosphoric acid. Absorbance was measured at 450nm with 630nm as the reference wavelength using an automated ELISA reader (Biotek Instruments, USA). The cut-off value was established as the OD obtained from healthy control sera plus three standard deviations (SD).

Indirect IgG ELISA was performed on all CHIKV immune sera at a dilution of 1:1000 with similar incubation conditions and washing steps (4 times). The plate was coated with 250 ng of whole virus antigen or 100 ng of rE2 and blocked with 3% BSA-0.05% PBST. Diluted sera were added to the plate and the bound antibodies were detected by addition of rabbit anti-human IgG-HRP (Dako Cytomation, cat. no. P0214) at 1:5000 dilution.

### Western blot

The proteins were resolved with 12% SDS-PAGE under non-reducing or reducing conditions and electro-transferred onto a nitrocellulose membrane (GE Healthcare, Germany). The membrane was blocked with 10% skimmed milk in 0.05% PBS-Tween 20 (PBST). For IgM detection, the pooled sera were treated with RIDA RF-Absorbens (R-Biopharm, Germany) in 1% bovine serum albumin (BSA)-PBS prior to blotting. The immunoreactivity of recombinant CHIKV proteins and virus antigen were evaluated at 1:100 and 1:400 dilutions. The bound antigen-antibody complex was detected by goat anti-human IgM-HRP (KPL, USA, cat. no. 474–1003) at 1:5000 dilution in 1% BSA-0.05% PBST. The membrane was visualized by chemiluminescence (Bio-Rad, USA) and images were acquired by BioSpectrum AC imaging system (UVP, USA). Mouse anti-His tagged antibodies (Merck Millipore, USA, cat. no. 05–949) were included as the control.

### Serum neutralization assay

Seroneutralization was performed with a previously described immunofluoresence-based cell infection assay in BHK-21 cells [27, 35, 36]. CHIKV immune sera were heat-inactivated and serially diluted 2-fold (1:100 up to 1:6400 dilution) in 1× Dulbecco’s PBS (DPBS). The diluted sera were mixed with CHIKV which was pre-diluted with 2% FBS GMEM in equal volumes, and the cells were infected at a final multiplicity of infection (MOI) of 10. The virus-antibody mixture was incubated for 2 hours at 37°C to improve binding capacity and to prevent the trapping of immunoglobulins in the cryoprecipitate [37]. The mixture was then inoculated into 10^4^ cells in a 96-well CellCarrier-96 optic black plate (Perkin Elmer, EU) and further incubated for 1.5 hours at 37°C. The inocula were decanted and 2% FBS GMEM was added. The plate was fixed with 4% paraformaldehyde after 6 hours of incubation at 37°C, permeabilized with 0.25% Triton X-100 for 10 minutes, and immunostained using CHIKV monoclonal antibody clone B-D2(C4) [33] at 1 μg/ml followed by rabbit anti-mouse IgG-FITC (Thermo Fisher Scientific, USA, cat. no. 31561) at 1:100 dilution. Cell nuclei were counter-stained with DAPI. Fluorescence intensity was analyzed with a Cellomics High Content Screening ArrayScan VTI (Thermo Fisher, USA) at 5× magnification. Percentage of infectivity was calculated with the following equation: % infectivity = (mean average fluorescence intensity from serum sample/mean average fluorescence intensity from virus control) × 100. The neutralizing titer (NT_50_) was expressed as the serum dilution that reduced infectivity by 50% using non-linear regression fitting in GraphPad Prism 5. For non-converged regressions, the neutralizing titer was set to 1.

To assess the IgM neutralization activity, the human IgG antibody from heat-inactivated sera was first precipitated with RIDA RF-Absorbens (R-Biopharm). The absorption buffer was prepared in 1:10 dilution in 1× DPBS and serum was diluted 2-fold with absorption buffer from 1:100 to 1:1600. The IgG antibody from samples was precipitated at 37°C for 30 min prior to mixing with CHIKV. The cells were rinsed with 1× DPBS at the end of cell-virus mixture incubation prior to replenishment with maintenance medium. For determination of neutralizing activity solely due to IgG, the heat-inactivated sera were treated with 0.1M dithiothreitol (DTT) (Life Technologies) to a final concentration of 5mM, to inactivate IgM, and were incubated at 37°C for 1 hour prior to dilution from 1:100 to 1:6400.

For seroneutralization using chimeric viruses, heat-inactivated serum was first treated to obtain anti-CHIKV IgM and IgG independently. Diluted sera were mixed with viruses pre-diluted with 2% FBS GMEM, with infection performed at an MOI of 50, followed by the steps described above. After 7 hours of incubation at 37°C, the plates were fixed and counter-stained with DAPI prior to acquisition of zsGreen fluorescence.

### Statistical analysis

Data are presented as means ± standard deviation (SD) or means ± standard error of the mean (SEM). Differences between groups and controls were analyzed using appropriate statistical tests, as stated in the figure labels. A *P*-value of <0.05 was considered significant. All the statistics were performed with GraphPad Prism 5.

## Results

### Appearance of neutralizing antibodies, particularly IgM isotype is associated with a reduction of viremia

**Fig 1:**
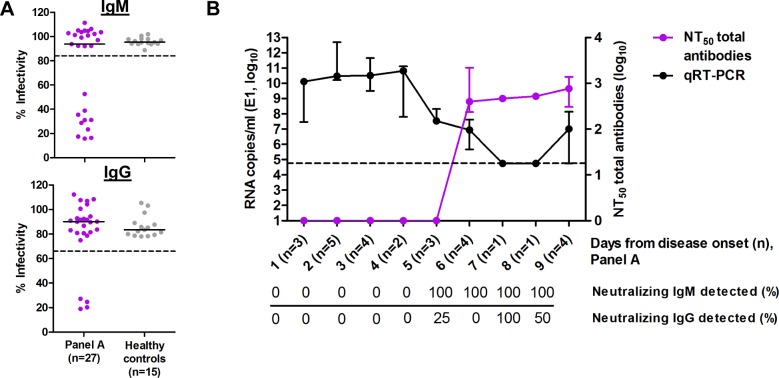
The appearance of CHIKV-specific neutralizing antibodies is associated with a reduction of viremia. (A) Seroneutralization was performed on sera from panel A which had known viral loads. Experiments were performed in triplicate at 1:100 dilution for detection of neutralizing IgM and IgG. The dotted lines represent the cut-off value determined from the mean—SD values from healthy control sera. (B) Neutralization titers (NT_50_) of total antibodies and previously-determined viral loads [26] of serum samples (panel A) collected at different times of disease onset were plotted. Each point indicates the median of RNA copies/ml targeting E1 and NT_50_, with the inter-quartile range. The dashed line indicates the limit of quantification (1 log_10_ RNA copies/reaction or 4.76 log_10_ RNA/ml) of the E1 positive-strand PCR assay [26].

The IgM and IgG titers were measured after IgG precipitation or IgM inactivation steps to ensure that the neutralizing roles of IgM and IgG were examined independently. The efficacies of IgG precipitation and IgM inactivation were 96% and 80%, respectively (S1 Fig). To understand the relationship between viremia and the appearance of neutralizing antibodies at different days of disease onset, viremic serum samples with known viral load were examined for the presence of neutralizing antibodies. Out of 27 viremic samples in panel A, 10 samples had neutralizing IgM, of which 4 samples had accompanying neutralizing IgG (Fig 1A). The decrease in viral load corresponded to the rise of neutralizing antibodies starting from day 6 after disease onset (Fig 1B). Neutralizing IgM was detected in all immune sera from day 6 onwards; however, there was variation in neutralizing IgG detected within the similar period from days 6 to 9.

### Anti-CHIKV IgM provides a short period of protection during the early phase of infection up to day 10

**Fig 2:**
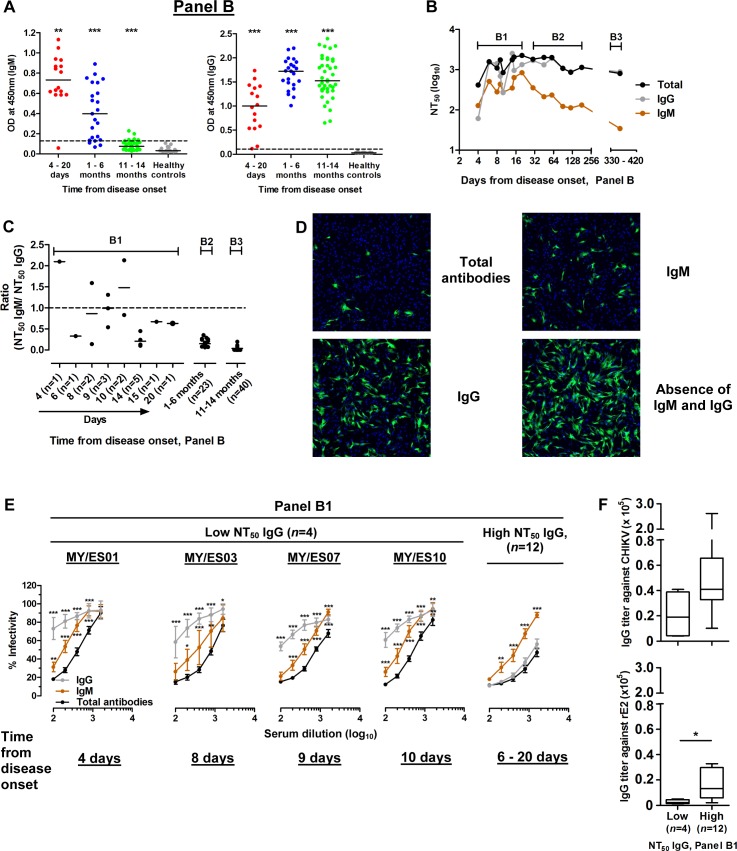
Anti-CHIKV IgM provides a short period of protection up to day 10 during early phase of infection. (A) Anti-CHIKV IgM and IgG were measured by ELISA. Samples were categorized by time from disease onset. **P<0.01, ***P<0.001, Mann-Whitney U test compared to healthy controls. The dotted lines represent the cut-off value. All the samples were within the detection limit except one. (B) The neutralizing titers of IgM, IgG and total antibodies were plotted by days from disease onset. Each point indicates the median of NT_50_. (C) Ratios of NT_50_ IgM over NT_50_ IgG were calculated and plotted for serum samples by times from disease onset (panel B). A ratio of 1 indicates equal strength of NT_50_ IgM over NT_50_ IgG. (D) Representative immunofluorescent microscopic images of CHIKV-infected cells incubated with 1:100 diluted serum under different treatment conditions. Objective magnification: 5× from 1 field of view. The serum sample used was from panel B1, collected 4 days post-onset of illness, with IgM and IgG titers of 20480 and 4096, respectively. (E) Panel B1 was divided into groups with either high or low NT_50_ IgG. Neutralization of virus infectivity due to IgM or IgG was compared to neutralizing capacity due to total antibodies. Results are expressed as percentage of virus control. *P<0.05, **P<0.01, ***P<0.001 by two-way ANOVA with Bonferroni multiple comparisons test. Data are presented as mean ± SD for individual samples with low NT_50_ IgG and mean ± SEM for 12 samples with high NT_50_ IgG. (F) Panel B1 was divided into groups with either high or low NT_50_ IgG ratio. IgG titers against CHIKV and rE2 were measured. *P<0.05, Mann-Whitney U test. The lines in the middle of the boxes indicate medians; the upper and lower boundaries of the box indicate inter-quartile ranges; and the whiskers indicate ranges of values.

The relative contributions of IgM and IgG towards neutralization were further characterized using the 79 serum samples from panel B. Anti-CHIKV IgM and IgG seroreactivities were analyzed using capture IgM and indirect IgG ELISA. Serum samples were categorized by collection time from disease onset, as either 4–20 days, 1–6 months, or 11–14 months. The anti-CHIKV IgM titers peaked during the early phase of infection (days 4–20) and waned over time (Fig 2A); IgM was detectable in most samples at 1–6 months, but was mostly undetectable by 11–14 months. Anti-CHIKV IgG titers rose from days 4–20 and were sustained up to 11–14 months (Fig 2A). The neutralizing titers demonstrated similar patterns as the antibody titers, with the neutralizing IgM waning over time while neutralizing IgG was sustained up to 11–14 months (Fig 2B). IgG generally contributed the most to neutralizing activity, but in 4 out of 16 samples from panel B1 (collected at days 4, 8, 9 and 10), the ratio of NT_50_ IgM/NT_50_IgG was more than 1, indicating a predominant role for neutralizing IgM (Fig 2C). These samples demonstrated that IgM plays a major neutralizing role to inhibit virus infection in the presence of low IgG titers (Fig 2D). The inactivation of IgM and precipitation of IgG in immune sera resulted in major loss of neutralizing activity (> 75% relative to virus control). Panel B1 was separated into 2 groups of low and high NT_50_ IgG for further analysis, based on Fig 2C. For panel B1 samples with low NT_50_ IgG, comprising the 4 samples for which neutralizing IgM was predominant, the individual contributions of IgM or IgG towards neutralization were significantly lower than the total antibodies neutralizing capacity (Fig 2E). This indicates that the overall neutralizing capacity was achieved with the combined presence of IgM and IgG acting in a complementary manner. As for panel B1 samples with high NT_50_ IgG, the total neutralizing capacity was similar to neutralizing capacity of IgG alone, with no significant additional effect of IgM (Fig 2E). Both low and high NT_50_ IgG groups had similar IgG titers against the whole virus antigen; however, the high NT_50_ IgG group had higher IgG titer against rE2 (Fig 2F). These results suggest that anti-CHIKV IgM provides a short period of protection during the early phase of infection for a duration of up to 10 days (varying between patients), before the mounting of an effective and strong anti-CHIKV IgG response targeting E2 glycoprotein.

### Anti-CHIKV IgM preferably targets epitopes on E1-E2 glycoproteins

**Fig 3:**
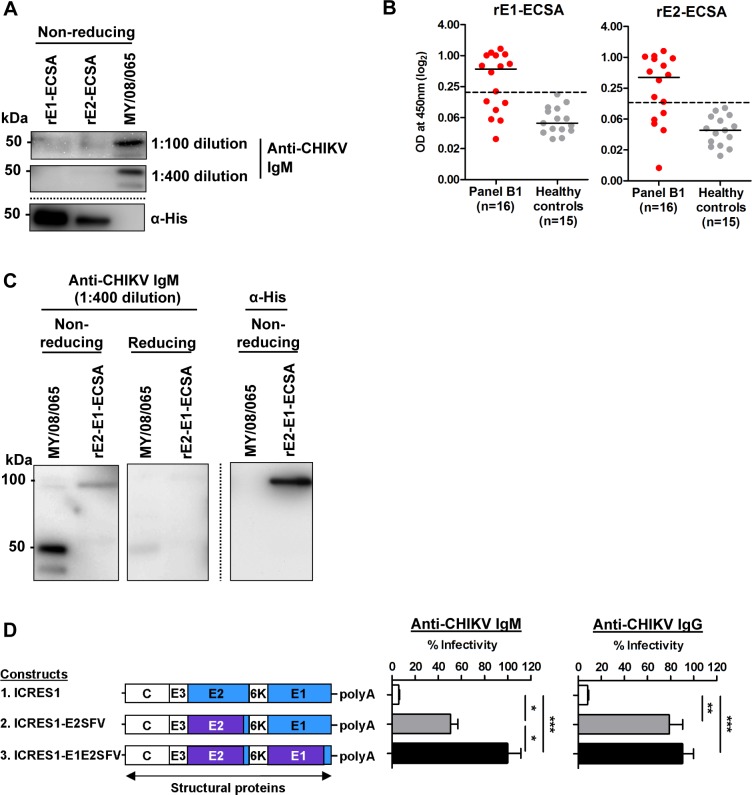
Anti-CHIKV IgM preferably targets epitopes on E1-E2 glycoproteins. (A) IgM immunoblotting was performed using approximately 1 μg of recombinant protein (E1 and E2) and 2.5 μg of whole virus antigen under non-reducing conditions at serum dilutions of 1:100 and 1:400 using pooled serum samples from panel B1. Mouse anti-His (α-His) was used as a control. (B) IgM reactivity against rE1 and rE2 in capture ELISA was investigated. The experiments were performed at 1:100 serum dilution. The dotted lines represent the cut-off value. (C) Immunoblotting was performed under non-reducing and reducing conditions at a serum dilution of 1:400 against fusion recombinant E2 and recombinant E1 glycoprotein (rE2-E1-ECSA). (D) Schematic diagram showing the chimeras used in seroneutralization with comparison of infectivity using pooled serum samples from panel B1. Data are presented as means ± SD from 4 independent experiments at a serum dilution of 1:100. *P<0.05, **P<0.01, ***P<0.001, Kruskal-Wallis test.

To further understand the neutralization characteristics of IgM, the target epitopes of IgM were investigated using the serum samples from panel B1 against the individual structural envelope glycoproteins. Immunoblotting analysis was performed under non-reducing conditions. At 1:100 serum dilution, anti-CHIKV IgM poorly recognized rE1 and rE2 glycoproteins with undefined bands, but strongly bound to the whole virus antigen, with a band of 50 kDa consistent with E1 or E2 (Fig 3A). At higher dilution (1:400), the recognition was only retained against the whole virus antigen, but not against rE1 or rE2 glycoprotein. The poor reactivity of IgM on Western blot against rE1 and rE2 was supported by capture ELISA (Fig 3B), which showed that more samples had below detectable IgM responses to either rE1 (n = 6 samples) or rE2 (n = 5) compared to whole virus antigen (n = 1, Fig 2A). Notably, 4 serum samples with below detectable IgM responses against rE1 or rE2 were from the same patient, yet had detectable IgM against the whole virus. This indicates that IgM can recognize epitopes on rE1 or rE2, but possibly binds to a combination of both glycoproteins. To determine the importance of epitopes resulting from interactions between E1 and E2 glycoproteins, immunoblotting was performed using a fusion E1-E2 glycoprotein as an antigen (rE2-E1-ECSA). At 1:400 serum dilution, IgM recognized this fusion protein under non-reducing conditions (Fig 3C) in contrast to individual rE1 or rE2 proteins (Fig 3A). The recognition by IgM was diminished when the fusion protein was subjected to reduction by DTT, which would lead to loss of conformation. To further verify the target epitopes of IgM, a neutralization assay was performed with previously constructed chimeras which had the ecto-domain regions of the CHIKV E2 and E1-E2 glycoproteins swapped with those of SFV [27]. Another parallel experiment was carried out to compare the target epitopes of early IgG. Loss of neutralization was expected as E1 and E2 are known to contain the main neutralizing epitopes for CHIKV. Loss of neutralization by both IgM and IgG was observed against the chimera with SFV E2 (Fig 3D). Using the chimera with SFV E1-E2 resulted in a significant additional loss of neutralization activity of IgM, but not of IgG (Fig 3D). This shows that neutralizing IgM targets epitopes on both E2 and E1-E2 glycoproteins, while the early neutralizing IgG mainly targets epitopes on E2. Taken together with the results from antibody binding studies and neutralization, IgM preferably recognized epitopes spanning E1-E2 glycoproteins, rather than epitopes on individual E1 or E2 glycoprotein.

## Discussion

Neutralizing antibodies in viral immunity provide protection by clearing viremia. Other effector functions of IgM and IgG are complement activation, opsonization of antigens for phagocytosis and antibody-dependent cell-mediated cytotoxicity [38, 39]. Neutralizing IgG in CHIKV infection, which persists for a long period, has been studied [11, 13, 14], particularly from the viewpoint of eliciting protective immunity by vaccination. However, the role of anti-viral IgM, which appears earlier and is normally present for only up to 3 months, is less well understood.

In this study, we defined the independent roles of anti-CHIKV IgM and IgG from immune sera, and showed that both antibody isotypes have neutralizing characteristics similar to previously reported humanized/mouse monoclonal antibodies [40–45]. In our cohort of acutely infected patients, anti-CHIKV IgM can be detected as early as day 4 (panel B1), and is present in most cases by day 6 (panel A), suggesting that the high valency of IgM could be important in reducing viremia before the production of robust neutralizing IgG with high affinity. A rapid decrease of viral load at day 5 was seen in the absence of detectable neutralizing antibodies. While it is likely at this point that there were low levels of antibodies below the limit of detection of the neutralization assay, there may also be a role for the innate immune response, involving the activation of cytokines and NK cells prior to full development of the adaptive immune response [46–49]. We noted that in rare cases (n = 7), IgM can persist in serum at 11–14 months, similar to reports in La Réunion and Indonesia [50–52]. Studies have described an association of unusually persistent IgM with chronic arthralgia, destructive arthritis and neurological complications [53–56]. In chronic joint disease, the persistent IgM may be in response to occult viral persistence, as CHIKV RNA has been detected in joint tissue in humans [47] and in mice [57], but the pathophysiological significance of this remains to be studied. In our study, IgM demonstrated neutralizing activity similar to IgG, albeit with a weaker neutralizing effect compared to IgG at higher serum dilutions. It will be useful to further evaluate anti-CHIKV IgM as a prognostic marker and to investigate the functional role of IgM from patients with persistent joint disease [58].

Between days 4–10, there was individual variation in the relative contributions of IgM and IgG to overall neutralization capacity, with a few patients displaying predominant IgM and most others having predominant IgG. After day 10 from disease onset, IgM contributes minimally to overall neutralizing activity, as neutralizing IgG plays the dominant role. During this period of seroconversion (day 4 to 10), strong overall neutralizing capacity can be achieved with synergistic binding action of IgM and IgG against infectious virus (Fig 2D). The early IgG response, but not IgM, targets a well-characterized linear epitope E2EP3 on the N-terminus of E2 glycoprotein, similar to linear epitope LP1 in our previous study [27, 35, 59]. We carried out a supplementary experiment to demonstrate the complementary activities of neutralizing IgM and specific anti-LP1 IgG (S1 Text). This reiterates the importance of specific early IgG acting in synergy with IgM (S2A Fig). The complementary effect of rabbit anti-LP1 IgG was dose-dependent, while the addition of non-neutralizing IgG to IgM did not have any effect on overall neutralizing capacity (S2B Fig). The timing of appearance and the amount of IgM and IgG may have significant impact on clinical outcome, and it has been shown that early neutralizing IgG3 response has been associated with faster viral clearance and reduced risk of persistent arthralgia [60]. For the clinical use of therapeutic antibodies, optimal epitope selection and relative proportions of IgM and IgG should be carefully determined to ensure the optimum synergistic effect instead of competitive binding.

There has been no prior study of IgM epitopes for CHIKV. Current commercial diagnostic serology kits to detect IgM in acute samples showed poor sensitivity when using recombinant E1 glycoprotein alone as the antigen, showing the importance of epitopes on E2 [61–68]. This was supported by our study, in which we showed that IgM recognizes conformation-dependent and reduction-sensitive epitopes on the E1-E2 fusion glycoprotein. This finding provides the basis for development of an optimized native antigen for reliable IgM detection [69–71].

Development of vaccine candidates also requires sufficient understanding of antibody responses to enable design of assays that reflect vaccine effectiveness. Our study offers several relevant insights. IgG ELISA is useful as it correlates with total neutralizing antibodies, which are predominantly IgG and are correlated with protection against symptomatic CHIKV [72]. However, antibody responses against different strains/genotypes might differ slightly, and further evaluation of the most suitable antigen is warranted [27]. In contrast, although IgM assays may theoretically be a useful indicator of early vaccine response, they are less reliable because the magnitude of the neutralizing IgM response varies between individuals and may not correlate with neutralizing IgG in the very early stages of infection (S3 Fig), and the IgM contributes minimally to total neutralizing capacity after day 10.

Our key findings were supported by additional study of acute and early convalescent samples from a 1999 outbreak of CHIKV (Asian genotype) in Klang [73, 74]. The complementary action of neutralizing IgM with IgG during the early phase of infection was shown (S4A Fig), and IgM recognized epitopes of the E1-E2 fusion glycoprotein (S4B Fig).

In conclusion, CHIKV-infected individuals develop neutralizing anti-IgM and anti-IgG during the early phase of infection, which mediate the clearing of viremia. Neutralizing IgM is particularly important up to day 10 of infection, when it acts in a complementary manner with the early IgG, after which a robust neutralizing IgG response consistently predominates. Anti-CHIKV IgM preferably recognizes epitopes on the CHIKV surface E1-E2 glycoproteins rather than E1 or E2 individually, which has implications for the design of diagnostic IgM assays.

## Supporting information

S1 Fig Efficacy of IgG precipitation and IgM inactivation in CHIKV immune sera. (A) ELISA to whole virus antigen was carried out to evaluate the efficacy of IgG precipitation from pooled acute sera at 1:100 dilution. The IgM level was maintained after treatment. Data are presented as mean + SD (n = 3). **P<0.01, Mann-Whitney U test. (B) ELISA to whole virus antigen was carried out to evaluate the efficacy of IgM inactivation by DTT from pooled acute sera at 1:100 dilution. The IgG level was maintained after treatment. Data are presented as mean + SD (n = 3). **P< 0.01, Mann-Whitney U test.
(PDF)

S2 Fig Complementary neutralizing activities of IgM and anti-LP1 IgG. (A) The addition of LP1 antibody at 0.5 μg/ml improved the overall neutralizing capacities in the presence of IgM. Data are presented as means ± SD. Dotted lines represent the infectivity with 0.5 μg/ml LP1 antibody (IgG) in the absence of IgM. (B) The complementary effects of neutralizing IgM and IgG antibodies was also shown with different concentrations of neutralizing anti-LP1 IgG at 1 and 3 μg/ml, but not with non-neutralizing IgG (mouse monoclonal antibody F-G6(F6) and rabbit antibody E2dA, which target linear epitopes on the surface of domain A, E2 glycoprotein). Dark green and purple dotted lines represent the infectivity with LP1 antibody treatment at 1 and 3 μg/ml, respectively, in the absence of IgM. Serum MY/ES10 was used in this experiment. All experiments shown were performed in triplicate from 1:100 to 1:1600 dilutions. Results are expressed as percentage of virus control. *P<0.05, **P<0.01, ***P<0.001 by two-way ANOVA with Bonferroni multiple comparisons test. Data are presented as mean ± SD.
(PDF)

S3 Fig Correlation between NT_50_ IgM and NT_50_ IgG. The correlation between NT_50_ IgM and NT_50_ IgG was assessed for different serum panels using Spearman’s rank correlation coefficients (ρ). *P*-values are shown; *ns*, not significant. Four samples from panel B1 were excluded from analysis as they were collected early within the seroconversion period, and NT_50_ IgM was predominant over NT_50_ IgG.
(PDF)

S4 Fig The used of archived serum samples from 1999 outbreak to study the neutralizing antibodies. (A) Neutralization of virus infectivity due to IgM or IgG was compared to neutralizing capacity due to total antibodies. Results are expressed as percentage of virus control. *P<0.05, **P<0.01, ***P<0.001 by two-way ANOVA with Bonferroni multiple comparisons test. Data are presented as mean ± SD for individual samples with low NT_50_ IgG and mean ± SEM for 11 samples with high NT_50_ IgG. (B) Schematic diagram showing the chimeras used in seroneutralization with comparison of infectivity using pooled serum samples. Data are presented as means ± SD from 4 independent experiments at a serum dilution of 1:100. *P<0.05, **P<0.01, ***P<0.001, Kruskal-Wallis test.
(PDF)

S1 Text Supplementary materials and methods. (DOCX)
