# Supplementary figures and images for: The neutralizing role of IgM during early Chikungunya virus infection
Source: PLoS One. 2017 Feb 9;12(2):e0171989. doi: 10.1371/journal.pone.0171989 (PMC5300252; doi:10.1371/journal.pone.0171989)

# S1 Fig

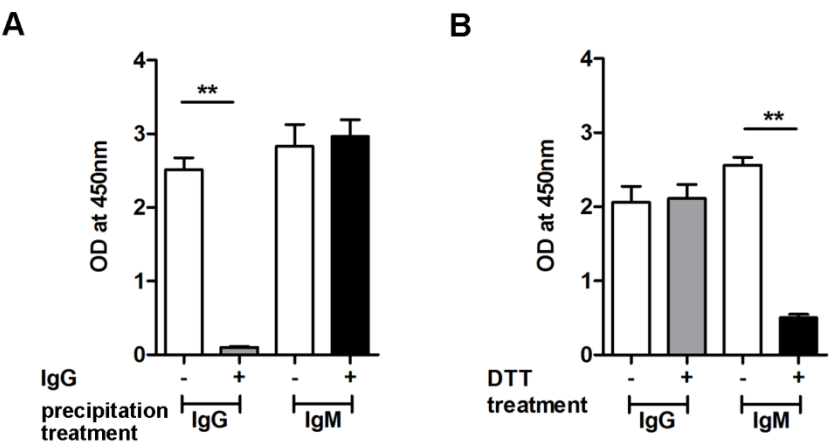

Supplement: S1 Fig — (A) ELISA to whole virus antigen was carried out to evaluate the efficacy of IgG precipitation from pooled acute sera at 1:100 dilution. The IgM level was maintained after treatment. Data are presented as mean + SD (n = 3). **P<0.01, Mann-Whitney U test. (B) ELISA to whole virus antigen was carried out to evaluate the efficacy of IgM inactivation by DTT from pooled acute sera at 1:100 dilution. The IgG level was maintained after treatment. Data are presented as mean + SD (n = 3). **P< 0.01, Mann-Whitney U test. (PDF) [file pone.0171989.s001.pdf]

# S2 Fig

A

Panel B1

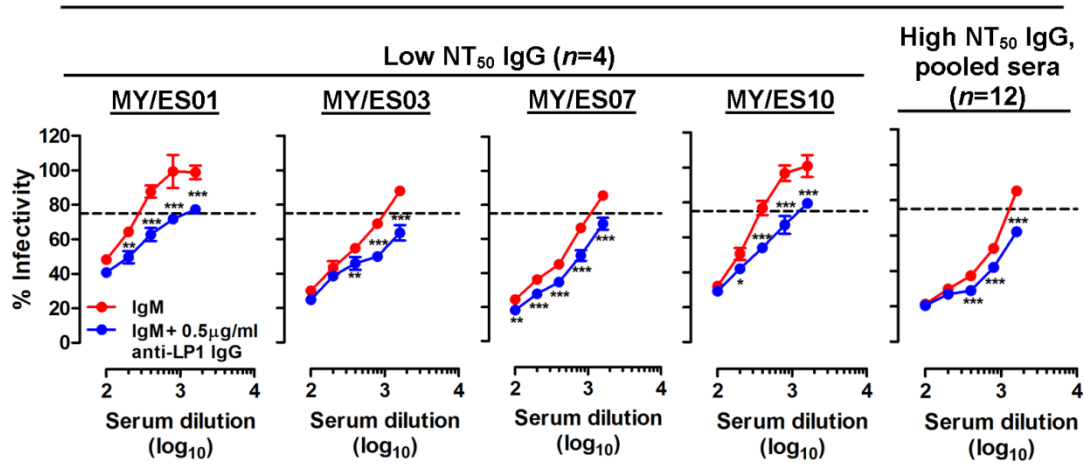

B

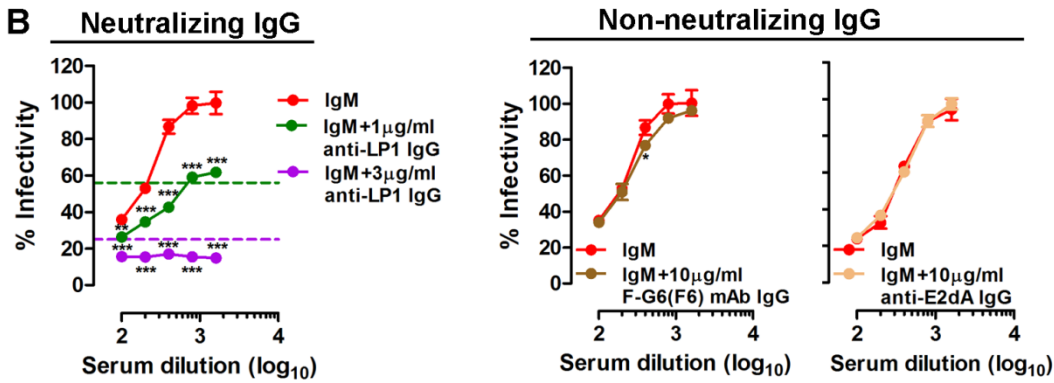

Supplement: S2 Fig — (A) The addition of LP1 antibody at 0.5 μg/ml improved the overall neutralizing capacities in the presence of IgM. Data are presented as means ± SD. Dotted lines represent the infectivity with 0.5 μg/ml LP1 antibody (IgG) in the absence of IgM. (B) The complementary effects of neutralizing IgM and IgG antibodies was also shown with different concentrations of neutralizing anti-LP1 IgG at 1 and 3 μg/ml, but not with non-neutralizing IgG (mouse monoclonal antibody F-G6(F6) and rabbit antibody E2dA, which target linear epitopes on the surface of domain A, E2 glycoprotein). Dark green and purple dotted lines represent the infectivity with LP1 antibody treatment at 1 and 3 μg/ml, respectively, in the absence of IgM. Serum MY/ES10 was used in this experiment. All experiments shown were performed in triplicate from 1:100 to 1:1600 dilutions. Results are expressed as percentage of virus control. *P<0.05, **P<0.01, ***P<0.001 by two-way ANOVA with Bonferroni multiple comparisons test. Data are presented as mean ± SD. (PDF) [file pone.0171989.s002.pdf]

# S3 Fig

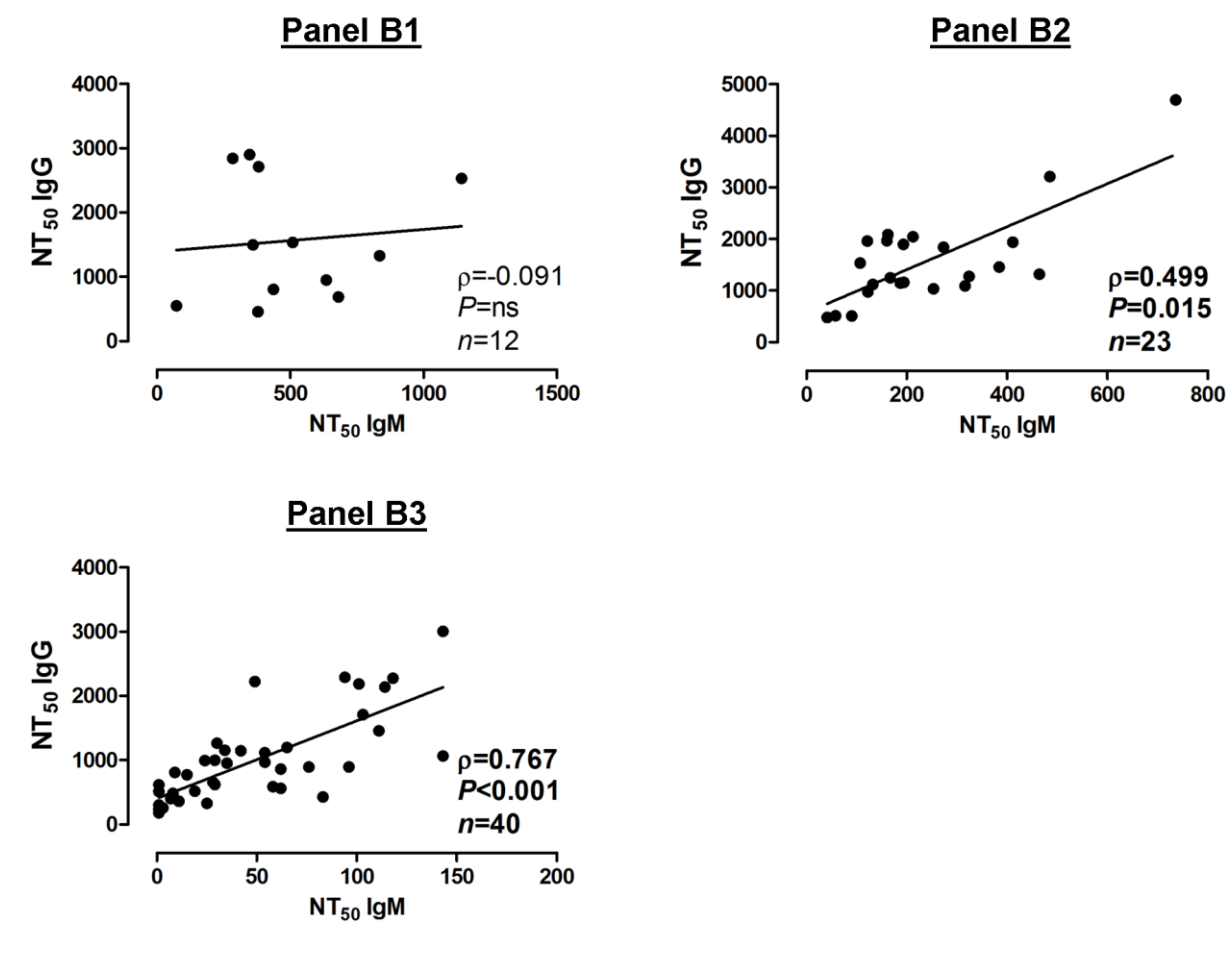

Supplement: S3 Fig — The correlation between NT50 IgM and NT50 IgG was assessed for different serum panels using Spearman’s rank correlation coefficients (ρ). P-values are shown; ns, not significant. Four samples from panel B1 were excluded from analysis as they were collected early within the seroconversion period, and NT50 IgM was predominant over NT50 IgG. (PDF) [file pone.0171989.s003.pdf]

# S4 Fig

A

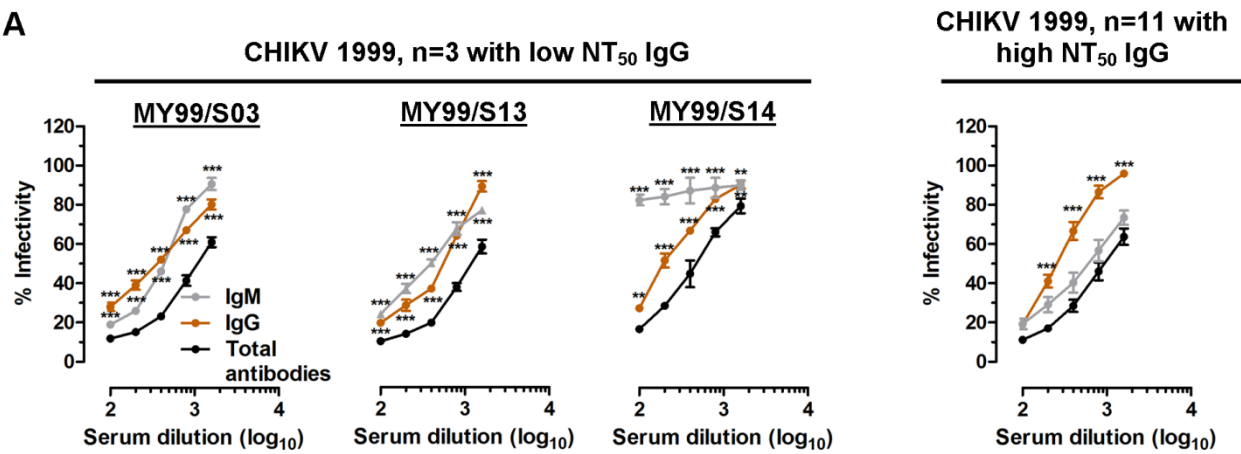

B

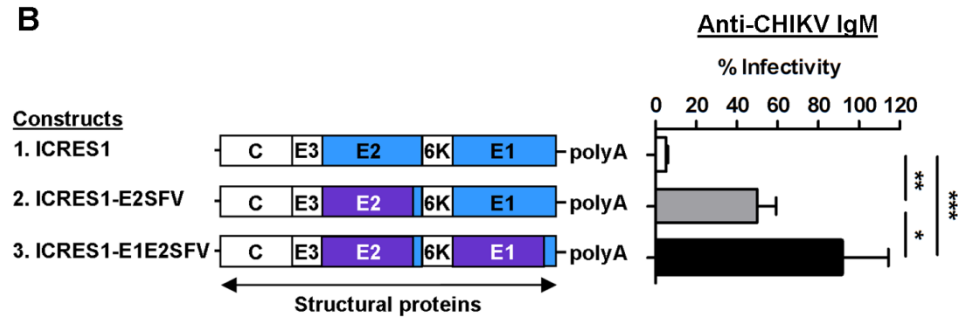

Supplement: S4 Fig — (A) Neutralization of virus infectivity due to IgM or IgG was compared to neutralizing capacity due to total antibodies. Results are expressed as percentage of virus control. *P<0.05, **P<0.01, ***P<0.001 by two-way ANOVA with Bonferroni multiple comparisons test. Data are presented as mean ± SD for individual samples with low NT50 IgG and mean ± SEM for 11 samples with high NT50 IgG. (B) Schematic diagram showing the chimeras used in seroneutralization with comparison of infectivity using pooled serum samples. Data are presented as means ± SD from 4 independent experiments at a serum dilution of 1:100. *P<0.05, **P<0.01, ***P<0.001, Kruskal-Wallis test. (PDF) [file pone.0171989.s004.pdf]
