# Supplementary material for: The neutralizing role of IgM during early Chikungunya virus infection
Source: PLoS One. 2017 Feb 9;12(2):e0171989. doi: 10.1371/journal.pone.0171989 (PMC5300252; doi:10.1371/journal.pone.0171989)
Supplement: S1 Text — (DOCX) [file pone.0171989.s005.docx]

**S1 Text**

**Supplementary materials and methods**

**Complementary neutralizing activities of IgM and anti-LP1 IgG antibodies**

Since linear epitope LP1 is a well-characterized key neutralizing linear epitope, polyclonal rabbit anti-LP1 (STKDNFNVYKATRPY) IgG was commercially prepared (GenScript, USA) and was used to study the interaction of neutralizing IgG in the presence of immune IgM. LP1 is similar to E2EP3, an immunogenic peptide (from an ECSA virus) previously reported to elicit neutralizing antibodies [[1](#_ENREF_1)]. The human IgG from heat-inactivated sera was precipitated with RIDA RF-Absorbens and 2-fold dilutions prepared from 1:100 to 1:1600. Anti-CHIKV IgM in sera was incubated with virus for an hour, followed by the addition of rabbit anti-LP1 antibody into each pre-diluted serum sample and incubation for another hour at 37°C. Neutralization assay was performed to verify the percentage of total neutralization capacity after addition of rabbit anti-LP1 antibody in the presence of anti-CHIKV IgM. Non-neutralizing mouse monoclonal antibody F-G6(F6) and rabbit polyclonal antibody anti-E2dA, which both target short linear epitopes (ADAERAGLFV and IKTDDSHDWTKLRY, respectively) in domain A of E2 [[2](#_ENREF_2)], were included as negative controls.

**References**

1. Kam YW, Lum FM, Teo TH, Lee WW, Simarmata D, Harjanto S, et al. Early neutralizing IgG response to chikungunya virus in infected patients targets a dominant linear epitope on the E2 glycoprotein. EMBO Mol Med. 2012;4: 330-343. doi: 10.1002/emmm.201200213. PMID: 22389221.

2. Chua CL, Chan YF, Sam IC. Characterisation of mouse monoclonal antibodies targeting linear epitopes on chikungunya virus E2 glycoprotein. J Virol Methods. 2014;195: 126-133. doi: 10.1016/j.jviromet.2013.10.015. PMID: 24134938.
